# A Systematic Review of SARS-CoV-2-Associated Hepatic Dysfunction and the Impact on the Clinical Outcome of COVID-19

**DOI:** 10.7759/cureus.26852

**Published:** 2022-07-14

**Authors:** Aleksandra Radivojevic, Anas A Abu Jad, Anvesh Ravanavena, Chetna Ravindra, Emmanuelar O Igweonu-Nwakile, Safina Ali, Salomi Paul, Shreyas Yakkali, Sneha Teresa Selvin, Sonu Thomas, Viktoriya Bikeyeva, Ahmed Abdullah, Prachi Balani

**Affiliations:** 1 Internal Medicine, California Institute of Behavioral Neurosciences & Psychology, Fairfield, USA; 2 Behavioral Neurosciences and Psychology, California Institute of Behavioral Neurosciences & Psychology, Fairfield, USA; 3 General Surgery, California Institute of Behavioral Neurosciences & Psychology, Fairfield, USA; 4 Medicine, California Institute of Behavioral Neurosciences & Psychology, Fairfield, USA

**Keywords:** coronavirus disease 2019, covid-19, transaminases, liver function tests, liver injury, liver failure, liver, sars-cov-2

## Abstract

Coronavirus disease 2019 (COVID-19) has rapidly spread across the globe since December 2019. The spectrum of clinical manifestations of COVID-19 ranges from mild to life-threatening forms. Alteration of hepatic function in COVID-19 is multifactorial. The objective of this systematic review is to assess the relationship between severe acute respiratory syndrome coronavirus 2 (SARS-CoV-2)-induced hepatic dysfunction and the clinical outcome in patients infected with COVID-19. We methodically explored several electronic databases (PubMed, PubMed Central, MEDLINE, and Google Scholar) in April 2022 using focused words and terms of medical subject headings for appropriate studies. We followed the Preferred Reporting Items for Systematic Reviews and Meta-Analyses (PRISMA) guidelines for conducting our systematic review. Hepatic dysfunction was identified as elevation of liver function tests (LFTs) above the upper limit of normal. The clinical outcome was described as a combination of mortality, intensive care unit (ICU) transfer, and the need for mechanical ventilation (MV). The initial search yielded a total of 7187 studies. After elimination of duplicates, exclusion of studies based on irrelevant titles and abstracts, comprehensive analysis of full-text formats, and evaluation of quality, a total of 16 studies were eligible to be included in our systematic review. In the 16 selected studies, there were 23,962 patients. The SARS-CoV-2 virus can negatively affect several organ systems by interacting with specific receptors widely expressed in the human body. A multifactorial etiology of hepatic dysfunction is observed in COVID-19. SARS-CoV-2 infection is associated with abnormal LFTs. Significantly higher mortality, ICU admissions, and requirement for MV are associated with LFT alterations. For this reason, patients infected with COVID-19 must have their hepatic function closely monitored.

## Introduction and background

Since the first reports of coronavirus disease 2019 (COVID-19) in Wuhan, China, the infection has spread rapidly throughout the world. Severe acute respiratory syndrome coronavirus 2 (SARS-CoV-2) is a novel beta-coronavirus that is responsible for this life-threatening disease in humans, with a fatality rate of 3.4% [1]. As of April 26, 2022, the World Health Organization (WHO) has confirmed 508,041,253 COVID-19 cases worldwide with 6,224,220 fatal outcomes [2].

Clinical presentation of COVID-19 is broad, ranging from a mild disease to a critical clinical condition. Even though most patients do not exhibit severe symptoms, the disease can also be fatal, with a mortality rate of 25% and more among those hospitalized in intensive care units (ICUs), mostly because of progressive respiratory failure and shock [3]. The most significant clinical manifestations are associated with lung damage. However, pathological clinical findings are present in other organs, including the liver, heart, pancreas, and kidneys. Angiotensin-converting enzyme 2 receptor (ACE2R) is expressed in numerous organs as the main viral entry [4,5]. In patients who have abnormal liver functions, SARS-CoV-2 may infect hepatocytes and bile duct cells due to the widespread ACE2R distribution in the body [4].

Deranged liver biochemistry in COVID-19 is multifactorial and associated with direct virus-induced cytotoxicity, immune liver dysfunction as a result of the inflammatory reaction (cytokine storm), and microthrombi formation in the hepatic parenchyma. Several of the drugs utilized to treat COVID-19-related symptoms also induce liver injury as well (e.g., remdesivir and tocilizumab). Furthermore, hypoxia, hemodynamic instability, and pre-existing chronic liver disease can also have detrimental effects on hepatic function [6]. It has been shown that abnormal liver function tests may be related to the severity of COVID-19 [7]. Moreover, hepatic dysfunction has been associated with adverse clinical outcomes and mortality in patients infected with SARS-CoV-2 [8].

Despite numerous studies that have been conducted until now, a definitive conclusion regarding liver dysfunction and the subsequent effect on the clinical outcome of patients infected with SARS-CoV-2 has not been established yet. Therefore, our systematic review aims to conduct an in-depth investigation of SARS-CoV-2-associated hepatic dysfunction and its impact on the clinical outcome of COVID-19.

## Review

Methods

This systematic review was conducted according to the Preferred Reporting Items for Systematic Reviews and Meta-Analyses (PRISMA) guidelines [9]. A comprehensive literature search had been performed up to April 10, 2022, utilizing electronic databases (PubMed, PubMed Central, MEDLINE, and Google Scholar) for data collection. We explored the databases by using the following terms of medical subject heading (MeSH) and keywords in combination to find relevant studies: “COVID-19” AND “SARS-CoV-2” AND “liver” AND “liver failure” AND “liver injury” AND “liver function tests” AND “transaminases.” We carefully examined the identified articles to exclude duplicates, scanned the titles and abstracts in the initial search for relevance, and retrieved full papers of potentially eligible studies for quality assessment. Our search strategy for electronic databases is demonstrated below in Table 1.

**Table 1:** Search strategy for electronic databases

Search strategy	Database	Number of articles
covid19 ("COVID-19"[Majr]) OR sars-cov-2 ("SARS-CoV-2"[Majr]) AND liver ("Liver"[Majr]) OR liver failure ("Liver Failure"[Majr]) OR transaminases ("Transaminases"[Majr]) OR liver function tests ("Liver Function Tests"[Majr])	PubMed, PubMed Central, MEDLINE	6767
“liver” AND “liver injury” AND “transaminases” AND “ liver function tests” AND “covid19”	Google Scholar	420

Inclusion Criteria

We included articles written in the English language that were published within the past two years, studies conducted on humans older than 19 years of age, and articles available in full-text format.

Exclusion Criteria

Grey literature, animal studies, studies not written in English, case reports, case series, overlapping studies, and studies involving pregnant patients were excluded.

Hepatic Dysfunction and Clinical Outcome

We defined hepatic dysfunction as the abnormal increment of any liver function test (LFT) value above the upper limit of normal (ULN), including serum aspartate aminotransferase (AST) over 40 U/L, serum alanine aminotransferase (ALT) over 50 U/L, alkaline phosphatase (ALP) > 140 U/L, gamma-glutamyl transferase (GGT) over 40 U/L, and total bilirubin (TBIL) > 21 μmol/L. The clinical outcome in this systematic review was identified as the combined endpoint of death, intensive care unit (ICU) admission, and the necessity for mechanical ventilation (MV).

Results

The initial inquiry identified 7187 studies based on our search criteria. After applying inclusion-exclusion criteria and eliminating duplicates, a total of 6672 articles were eliminated. Following the further selection of relevant articles based on title and appropriate abstract, we screened 99 full-text records. After establishing a 70% benchmark, a total of 74 studies were assessed for quality, and only 16 qualified after applying the quality assessment tools. We used the following means: Newcastle-Ottawa Scale (NOS) for observational studies, Appraisal Tool for Cross-Sectional Studies (AXIS), Assessment of Multiple Systematic Reviews (AMSTAR), and Scale for the Assessment of Narrative Review Articles (SANRA).

**Figure 1:**
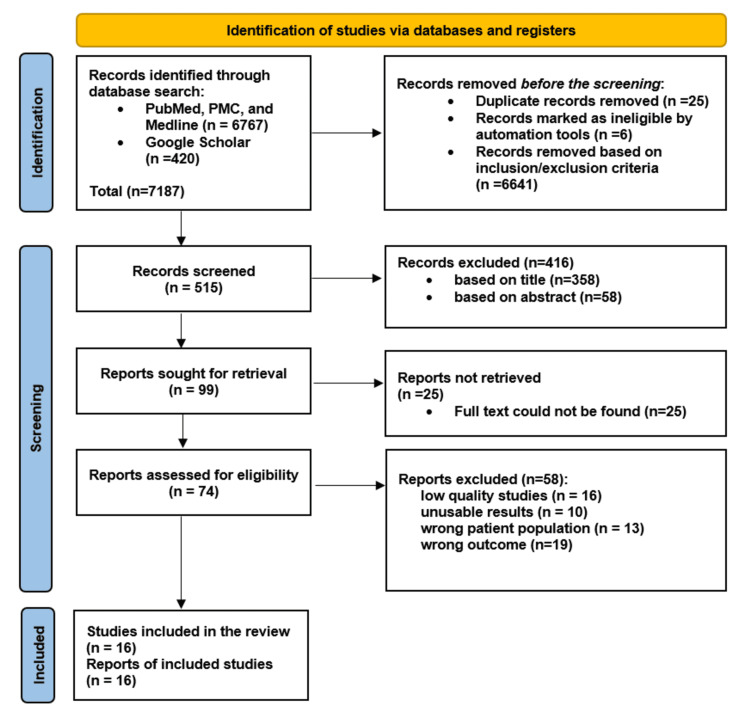
PRISMA (Preferred Reporting Items for Systematic Reviews and Meta-Analyses) flowchart presenting study selection strategy PMC: PubMed Central.

Our systematic review includes a total of 23,962 patients from 16 different studies, 13 retrospective cohort studies, two prospective cohort studies, and one cross-sectional study. Among the 16 articles, six are from Asia (India, China, Turkey, and Israel), four are from Europe (Croatia, Spain, France, and Italy), and three are from North America (New York, New Jersey, and a multicentric cohort study from 36 hospitals across North America) and three from South America. As demonstrated below, Figure 1 shows the study selection strategy for this systematic review [9].

Discussion

We conducted a systematic review of the literature on the association of SARS-CoV-2-induced hepatic dysfunction and the clinical outcome in COVID-19 patients, defined as a composite of mortality, ICU admission, and MV requirement.

Pathophysiology of COVID-19 Infection

The culprit of the COVID-19 pandemic, SARS-CoV-2-virus, is a positive, single-stranded RNA virus that belongs to the Coronaviridae family [10]. The viral genetic material is surrounded by a double-layered lipid membrane with incorporated spike protein, identified as S protein [11]. The S protein, which is a major glycoprotein, entails two subunits. The S1 subunit, with its receptor-binding domain (RBD), detects and attaches to the angiotensin-converting enzyme 2 receptors (ACE2R) expressed on the surface of the human host cell. The S2 subunit enables the fusion of the viral and target cell membrane [12]. Upon this merging, the type 2 transmembrane serine protease (TMPRSS2) found on the target cell cleaves ACE2R and activates S proteins [13]. As a result of S proteins' activation, host cell conformation is altered, facilitating viral entry, followed by the subsequent release of viral RNA and its transcription in the nucleus [11]. This initial stage of infection can further develop into the second phase of viral lung inflammation [14]. A crucial role in the development of lung injury, as the main location of the initial attack, is played by a set of pro-inflammatory molecules, i.e., cytokines. COVID-19 patients can develop an uncontrollable reaction of the immune system, driven by the enormous release of cytokines, the infamous cytokine storm, resulting in alveolar invasion by monocytes and macrophages [15]. It has been observed that more than 50% of patients infected with COVID-19 have an increased value of interleukin (IL)-6, which is a substantial mediator of the inflammatory response elicited by either infection or damage [16]. Additionally, several other cytokines are observed to be elevated as well, such as IL-2, IL-7, IL-10, and tumor necrosis factor-alpha (TNF-alpha) [14].

Although being dominantly expressed in the lower respiratory apparatus, especially in the alveolar type II cells, the type I integral membrane protein ACE2R can be found abundantly within the human body [17,18]. Therefore, SARS-CoV-2 can impact multiple organ systems, such as the gastrointestinal and hepatobiliary system, central nervous system, renal proximal tubular cells, liver, heart, bladder, and other organs [19].

**Figure 2:**
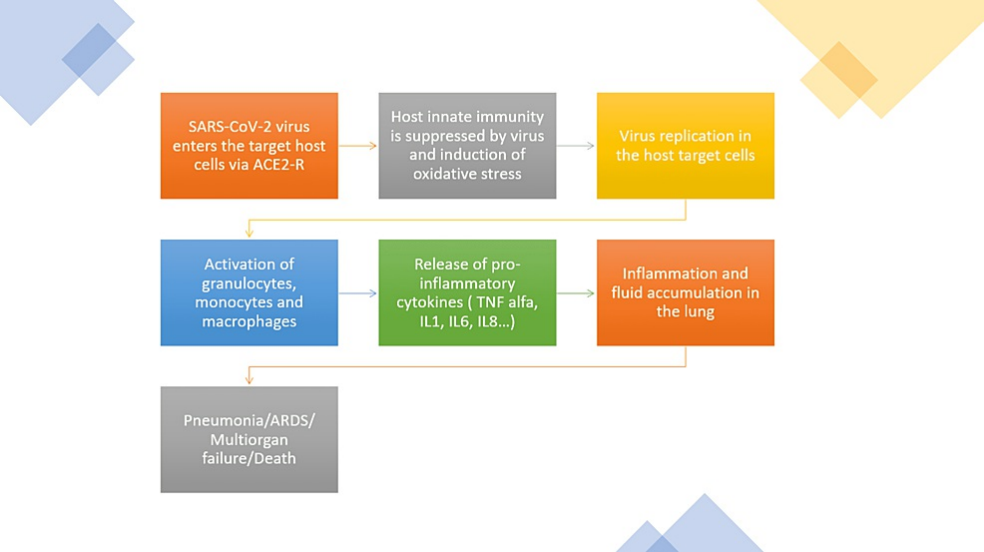
Schematic demonstration of COVID-19 pathophysiology ACE2R: angiotensin-converting enzyme 2 receptors; TNF-alpha: tumor necrosis factor-alpha; IL: interleukin; ARDS: acute respiratory distress syndrome.

Direct human-to-human contact is the main mode of spread of the SARS-CoV-2 virus, involving aerosolization of droplets via coughing and sneezing [17]. Infection with the SARS-CoV-2-virus leads to an array of clinical manifestations, from a mild self-restricting disease to a life-threatening multi-system involvement [20]. As shown below, Figure 2 demonstrates the pathophysiology of COVID-19.

Mechanism of Hepatic Damage in COVID-19

COVID-19-related hepatic damage refers to any liver damage occurring during the COVID-19 treatment or disease development, irrespective of the pre-existing hepatic condition [21].

The mechanisms involved in hepatic damage in COVID-19 patients are complex and multifactorial, including the direct cytopathological effect of the SARS-CoV-2 virus, immune hepatic dysfunction from dysregulated inflammatory reaction (the cytokine storm), and the appearance of microthrombi in the liver [21-24]. Additionally, tocilizumab and remdesivir are some of the many therapeutic agents used to treat symptomatic COVID-19 that can elicit hepatic damage. Furthermore, the impairment of hepatic function can also be caused by decreased oxygen blood supply, hemodynamic imbalance, and pre-existing hepatic conditions [21,22].

SARS-CoV-2 may target the liver due to similar expression of ACE2R on cholangiocytes to type 2 alveolar cells in the lower respiratory tract [4].

In a Chinese cohort of 657 patients, 32.3% of individuals suffering from a severe and critical form of COVID-19 presented with elevated GGT values in comparison to 19.3% of moderately ill individuals. In addition, abnormally high GGT and bilirubin values indicated that SARS-CoV-2 might be responsible for hepatic injury by damaging the cholangiocytes [21]. Davidov-Derevynko et al. found that elevation of aminotransferases was dominantly presented compared to ALP increment in their sample of 382 patients, suggesting a hepatocellular pattern of liver damage rather than a cholestatic one [22]. In a French study of 281 patients, the elevated level of GGT was the most common liver abnormality (25.3%), while a significant increase in ALP was less common. This was indicative of a cholestatic pattern of hepatic dysfunction [23]. Similarly, Bernal-Monterde et al. reported a strong association between GGT values and levels of ALP (r = 0.64, p < 0.001) and bilirubin (r = 0.19, p < 0.001) in hospitalized COVID-19 patients, thereby suggesting that a cholestatic pattern of liver damage is a substantial feature of participants with decreased survival rate [25].

During COVID-19 infection, there is a significant inflammatory response in the human body, leading to hypoxia and cytokine storm. Patients with critical illness may suffer hepatic injury and even hepatic failure [23]. Piano et al. argue the possibility of hepatic damage occurring as a result of an excessive and abnormal immune response of the human body against the SARS-CoV-2 virus. Furthermore, it has been stated that in COVID-19 patients with abnormal LFTs, the worse clinical course might be a consequence of either the virus spreading to at least one organ other than the lungs, such as the liver, or systemic inflammation. The aforementioned is reinforced with evidence of elevated levels of white blood cells, polymorphonuclear cells, ferritin, and C-reactive protein (CRP) in individuals with markedly altered liver biochemistry [24].

Similarly, a retrospective, single-center study of 540 patients from Spain reported that ferritin and high sensitivity C-reactive protein (hs-CRP) were significantly increased in participants who succumbed to COVID-19 during hospital occupancy. A significant association was observed between inflammatory biomarkers and liver biochemistry abnormalities, implicating that an excessive release of cytokines can contribute to liver damage, by inducing abnormal inflammatory reactions and subsequent multiorgan damage [25]. Additionally, Sobotka et al. found that substantially altered hepatic enzymes are more associated with severe inflammatory response and unstable hemodynamics than with SARS-CoV-2 affinity for the liver. This suggests that hepatic damage might be induced by a severe systemic response to the ongoing COVID-19 infection [26].

The respiratory insufficiency in COVID-19 patients leads to an impaired oxygen supply, which negatively impacts hepatic cells and contributes to hepatic damage [27]. Bernal-Monterde et al. demonstrated a negative correlation between aminotransferases and oxygen, which might originate from impaired oxygen delivery caused by respiratory infection [25].

**Figure 3:**
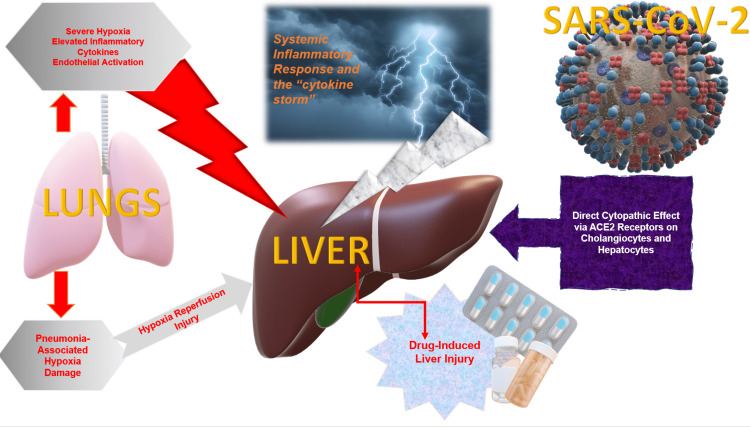
Possible mechanisms of hepatic damage in patients infected with SARS-CoV-2 virus ACE2: angiotensin-converting enzyme 2.

Vulnerability to SARS-CoV-2 hepatic damage may be more accentuated in individuals with pre-existing hepatic conditions [28]. Hepatic damage may also result from therapeutic agents used to manage COVID-19, which is indicated by the existence of microvesicular steatosis and hepatic inflammation during COVID-19 infection [29]. Wang et al. found that systemic glucocorticoids were administered in a significant percentage of patients with hepatic damage, suggesting drug toxicity is an important factor in the development of hepatic damage [21]. Similarly, an Italian study of 565 participants showed that a new development of abnormalities in liver blood tests was commonly observed during hospitalization. These were attributable to the use of medication that might be hepatotoxic, such as acetaminophen, tocilizumab, lopinavir, ritonavir, and antibiotics [24]. Studies providing explanations regarding possible mechanisms of hepatic damage in patients infected with the SARS-CoV-2 virus are demonstrated below in Table 2 and Figure 3.

**Table 2:** Characteristics of studies explaining possible mechanisms of hepatic damage in COVID-19 patients AST: aspartate aminotransferase; ALT: alanine aminotransferase; ALP: alkaline phosphatase; GGT: gamma-glutamyl transferase; TBIL: total bilirubin; ULN: upper limit of normal; LFTs: liver function tests; LCT: liver chemistry tests; ICU: intensive care unit; ECMO: extracorporeal membrane oxygenation; hs-CRP: high sensitivity C-reactive protein; IL: interleukin; TNF-alpha: tumor necrosis factor-alpha.

No.	Author	Year	Type of study	Patients	Purpose of the study	Results	Conclusion
1.	Chaibi et al. [23]	2021	Retrospective single-center study	281	Examination of the prognostic value of abnormal liver function parameters in a French sample of 281 hospitalized COVID-19 patients.	Out of 281 patients with COVID-19, a total of 102 (36.3%) presented with abnormal liver function parameters. Abnormal increments in AST and ALT were significantly associated with a higher rate of transfer to ICU and overall mortality.	In addition to being significantly related to a poor prognosis, liver test alterations could also be significantly helpful in the early detection of severe COVID-19 disease.
2.	Davidov-Derevynko et al. [22]	2021	Retrospective single-center study	382	Among hospitalized COVID-19 patients to evaluate the prevalence, degree, and outcome of hepatic damage as well as to assess how COVID-19 affects individuals with the pre-existing hepatic condition.	ALT > ULN, 111 (42.5%); AST > ULN, 174 (66.4%); GGT > ULN, 120 (55.8%); ALP > ULN, 39 (15.1%); bilirubin > ULN, 27 (10.3%). The hepatocellular pattern of liver damage was dominantly observed, with AST higher than ALT in 76.7% of patients. Deceased patients had substantially increased AST/ALT ratio (1.98 (1.34-2.51),p < 0.0001).	Patients with previous liver conditions had higher mortality compared to patients without the previous liver condition (22 (6.8%) vs. 10 (16.7%), p = 0.01).
3.	Sobotka et al. [26]	2021	Retrospective multicentric cohort	1555	To investigate the prevalence and severity of liver test derangements in a population of 1555 hospitalized COVID-19 patients.	During hospitalization, 74% of 1555 patients developed ALT elevation, which was extreme (over 20x ULN) in 43(3%). Increment in ALT and ALP values was associated with transfer to ICU, need for mechanical respiratory support, ECMO, the utility of vasopressor medication, and longer hospital occupancy.	Hepatic enzyme derangements give the impression of being associated with mortality and longer hospital stays, most probably resulting from severe systemic disease and not SARS-CoV-2-associated hepatitis.
4.	Bernal-Monterde et al. [25]	2020	Retrospective single-center cohort	540	To evaluate altered liver biochemistry at the beginning and during hospitalization, and its prognostic aspect in COVID-19 patients.	On admission, altered LFTs over 2x ULN presented in 29.2% of individuals and were related to short-term mortality. In a total of 39.8% of patients, without LFTs alteration, a new-onset of LFTs elevation over 2x ULN was observed to correlate with the overall death rate.	COVID-19 patients with a moderate LFT change at admission showed an increased risk of mortality within one week of hospitalization. It has also been reported that new onset of LFTs alteration over 2x ULN is related to overall death in hospitalized COVID-19 patients.
5.	Piano et al. [24]	2020	Retrospective multicentric cohort	565	To examine the prevalence, the clinical features, and the influence of abnormal liver chemistry tests (LCT) in hospitalized patients with non-critical COVID-19 disease.	Among 565 patients, 329 (58%) had abnormal LCT. Patients with abnormal LCT experienced more severe disease, organ dysfunction, higher rate of ICU admission (20% vs. 8%; p < 0.001), need for artificial ventilation (14% vs. 6%; p = 0.005), and death (21% vs. 11%; p = 0.004), compared to those with normal LCT.	In COVID-19 patients, LCT abnormalities are common upon admission, are related to systemic inflammation and organ dysfunction, and are predictive of ICU transfer or mortality.
6.	Wang et al. [21]	2020	Retrospective cohort	657	Assessment of risk factors for hepatic injury occurrence, and clinical features of COVID-19 patients with hepatic injury.	Hepatic damage was observed in 42.2% of 657 participants with elevated ALT, 4.9% of individuals with elevated TBIL, and 24.4% of patients with elevated GGT. hsCRP (>10 mg/L): patients with liver damage (230/274, 83.9%) vs. without liver damage (220/334, 65.9%). Serum IL-2R, IL-6, and TNF-alpha were of higher values in patients with liver damage compared to those without liver damage.	Liver damage in patients infected with COVID-19 might be caused by an immune-mediated inflammatory reaction.

Association Between Abnormal LFTs and the Clinical Outcomes (Mortality, ICU Admission, and MV) in COVID-19 Patients

Patients infected with COVID-19 commonly experience LFT abnormalities [30]. Huang et al. showed that patients with an AST level over three times the ULN (>3x ULN) had the greatest risk of mortality and artificial ventilation. A significant association was also observed between these two adverse outcomes and elevated levels of ALP and TBIL [31].

In an Italian cohort of 565 patients, 58% had altered liver biochemistry. These patients demonstrated a greater frequency of ICU admission (20% vs. 8%, p < 0.001), need for artificial respiratory support (14% vs. 6%, p = 0.005), and death (21% vs. 11%, p = 0.004) when compared to the group with normal liver biochemistry. Therefore, as the study suggests, patients with altered liver biochemistry should be carefully monitored for possible detrimental clinical outcomes [24]. Similarly, Pastrovic et al. emphasized as the most significant result of their study a transparent relationship between deranged LFTs with adverse clinical outcomes, which include mortality, admission to the ICU, and the need for MV. A significantly higher frequency of detrimental outcomes was observed in individuals with hepatic injury (defined as an elevation of bilirubin and hepatic enzymes) in comparison to patients with isolated alteration of hepatic enzymes [32]. Interestingly, Sobotka et al. reported that mortality during hospital occupancy did not appear to be associated with the degree of ALT or ALP elevations on hospital admission. Nonetheless, extreme hepatic enzyme derangements (over 10-20x ULN) were associated with an increased risk of death. Furthermore, participants with moderate and severe ALT or ALP alteration experienced higher rates of ICU transfer and MV [26].

In a retrospective study from South America, 29.2% out of 298 patients had a moderate abnormality of liver enzymes (ALE) over two times the upper limit of normal (>2x ULN) on admission, which was found to be an independent risk factor for mortality at one-week hospital occupancy. Furthermore, the newly developed ALE > 2x ULN during hospitalization was significantly related to global mortality in hospitalized COVID-19 patients [33]. A prospective multicenter study from Brazil reported a significantly increased mortality risk among patients with moderately elevated aminotransferases levels (>2x ULN) on admission in contrast to the group with mild alterations (less than 2x ULN). However, TBIL elevation was not related to global in-hospital mortality (HR = 0.98, 95% CI: 0.93-1.04) [34]. Salık et al. observed a substantial relationship between deranged LFTs (AST, ALT, and TBIL over ULN) and hepatic injury (AST and/or ALT > 3x ULN and/or TBIL > 2x ULN) with an increased risk of death in critically ill patients infected with COVID-19 [35].

In a cross-sectional study, among 235 patients with hepatic dysfunction, 19.6% died, compared to a mortality rate of 2.1% in 335 individuals with normal hepatic function (p < 0.01) [36]. Satapathy et al. argue that the constellation of all four liver blood tests (AST, ALT, ALP, and TBIL) displays a better predictive value in mortality risk in contrast to isolated hepatic alterations [30].

Among 281 participants in a French cohort, 102 presented with hepatic dysfunction. Increased GGT value was the most frequent alteration, followed by an increase in AST (24.3%) and ALT (12.8%) values. Profound elevations in aminotransferases levels were associated with severe disease and had a higher rate of overall mortality (26.7% vs. 12.1%, p = 0.03) and ICU admission (40.0% vs. 6.0%, p < 0.0001). Similar findings were observed in patients with a cholestatic pattern of hepatic damage [23]. Makar et al. demonstrated a substantial relationship between liver damage and a higher risk of death, the requirement for ICU, and length of stay. The global mortality in this study was 18% (97/539), and among deceased individuals, 88.7% had liver damage [37]. In line with the previous study, 45.2% of 1611 COVID-19 patients presented with altered LFTs on admission in a large multicentric cohort from South America. They had a significantly higher mortality rate when compared to individuals with normal LFTs (18.7% vs. 12.2%, p < 0.0001) as well as more frequent transfers to ICU [38]. Furthermore, in a retrospective cohort study from Turkey, higher rates of ICU admission and mortality were observed in the group with increased transaminase levels in contrast to the group with normal transaminase readings (22.9% vs. 10.5% and 13.7% vs. 4.7%, respectively). These results highlight the prognostic significance of abnormal hepatic biochemistry on hospital admission in predicting the severity of COVID-19 [39].

Characteristics of studies depicting the relationship between abnormal LFTs and clinical outcomes in COVID-19 patients are presented below in Table 3.

**Table 3:** Characteristics of studies describing the association between liver function tests and clinical outcomes in COVID-19 patients AST: aspartate aminotransferase; ALT: alanine aminotransferase; ALP: alkaline phosphatase; GGT: gamma-glutamyl transferase; TBIL: total bilirubin; ULN: upper limit of normal; LCA: liver chemistry abnormalities; LDH: lactate dehydrogenase; LFTs: liver function tests; LCT: liver chemistry tests; ALE: abnormal liver enzymes; NR: not reported.

No.	Author	Year	Type of study	Patients	Liver function parameters	Abnormal liver function tests	Mortality	Intensive care unit admission	Need for mechanical ventilation
1.	Balderramo et al. [33]	2021	Retrospective multicenter study	298	AST, ALT, GGT, ALP, TBIL	No ALEx2 (n = 211) vs. ALEx2 (n = 87)	45 (21.5%) vs. 21 (24.1%), p = 0.62	99 (47.1%) vs. 38 (43.7%), p = 0.59	72 (34.1%) vs. 27 (31%), p = 0.61
2.	Chaibi et al. [23]	2021	Retrospective single-center study	281	AST, ALT, GGT, ALP	AST or ALT ≥ 2x ULN (n = 76) vs. AST or ALT < 2x ULN (n = 206); GGT ≥ 2x ULN (n = 71) vs. GGT < 2x ULN (n = 210)	20 (26.7%) vs. 25 (12.1%), p = 0.003; 9 (12.7%) vs. 36 (17.1%), p = 0.38	30 (40.0%) vs. 13 (6.3%), p < 0.0001; 22 (31.0%) vs. 21 (10.0%), p < 0.0001	NR
3.	Makar et al. [37]	2021	Retrospective cohort	539	AST, ALT, ALP, TBIL	Liver injury (n = 345) vs. no liver injury (n = 194)	86 (24.9%) vs. 11 (5.7%), p < 0.0001	86 (24.9%) vs. 7 (3.6%), p < 0.0001	NR
4.	Mendizabal et al. [38]	2021	Prospective cohort	1611	ALT, ALP, TBIL	Normal LFTs (n = 882) vs. abnormal LFTs (n = 729)	12.2% vs. 18.7%, p < 0.0001	150 (17.0%) vs. 220 (30.2), p < 0.0001	115 (13.0%) vs. 177 (24.3%), p < 0.0001
5.	Paštrovic et al. [32]	2021	Retrospective cohort	3812	AST, ALT, GGT, ALP, TBIL	Normal LFTs vs. abnormal LFTs	2497 (65.5%) vs. 1315 (34.5%)	2933 (76.9%) vs. 879 (23.1%)	3152 (82.7%) vs. 660 (17.3%)
6.	Salık et al. [35]	2021	Retrospective cohort	533	ALT, AST, TBIL	Normal LFTs (n = 256); abnormal LFTs (n = 231); liver injury (n = 46)	152 (59,4%); 165 (71,4%); 36 (78,3%), p = 0.004	NR	NR
7.	Pozzobon et al. [34]	2021	Prospective multicenter cohort	406	AST, ALT	AST ≥ 2× ULN vs. AST < 2× ULN; ALT ≥ 2× ULN vs. ALT < 2× ULN	AST: 30.7 (19.8-47.6) vs. 10.6 (7.8-14.4), p < 0.001; ALT: 27.7 (17.2-44.6) vs. 11.3 (8.4-15.1), p = 0.001	NR	NR
8.	Satapathy et al. [30]	2021	Retrospective multicentric cohort	10,856	AST, ALT, ALP, TBIL	No LCA (n = 3096, 28.5%); mild to moderate LCA (n = 6933, 63.9%); severe LCA (n = 827, 7.6%)	No LCA is the reference; HR: 1.56 (1.38-1.76), p < 0.001; HR: 1.87 (1.52-2.30), p < 0.001	NR	NR
9.	Singhai et al. [36]	2021	Cross-sectional	678	AST, ALT, TBIL	Abnormal LFTs (n = 265, 44.2%) vs. normal LFTs (n = 335, 55.8%)	52 (19.6%) vs. 7 (2.1%), p < 0.01	NR	NR
10.	Sobotka et al. [26]	2021	Retrospective multicentric cohort	1555	ALT, ALP	ALT: normal vs. 1-3x ULN; ALP: normal vs. 1-2x ULN	ALT: 19% vs. 17%, p < 0.001; ALP: 14% vs. 28%, no p-value	ALT: 31% vs. 42%, p < 0.001; ALP: 38% vs. 63%, p < 0.001	ALT: 2% vs. 29, p < 0.001; ALP: 25% vs. 53%, p < 0.001
11.	Medetalibeyoglu et al. [39]	2020	Retrospective single-center cohort	554	ALT, AST, ALP, GGT, LDH, bilirubin	AST-ALT ≤ 40 (n = 401) vs. AST-ALT > 40 (n = 153)	19 (4.7%) vs. 21 (13.7%), p < 0.001	42 (10.5%) vs. 35 (22.9%), p < 0.001	NR
12.	Piano et al. [24]	2020	Retrospective multicentric cohort	565	AST, ALT, ALP, GGT, bilirubin	Normal liver function tests (n = 236) vs. abnormal liver function tests (n = 329)	26 (11%) vs. 68 (21%), p = 0.004	18 (8%) vs. 65 (20%), p < 0.001	15 (6%) vs. 47 (14%), p = 0.005
13.	Huang et al. [31]	2020	Retrospective Cohort	675	ALT, AST, TBIL	ALT: 40-120 U/L and over 120 U/L; AST: 40-120 U/L and over 120 U/L; TBIL: 21-63 µmol/L and over 63 µmol/L	ALT: HR 3.18 (1.05-9.67), p = 0.0412; 10.50 (2.94-37.48), p = 0.0003; AST: HR 9.16 (2.40-34.92), p = 0.0012; 19.27 (4.89-75.97), p <0.0001; TBIL: HR 5.29 (2.00-14.01), p = 0.0008; 7.01 (1.24-39.63), p = 0.0275	NR	ALT: HR: 7.02 (2.74-18.02), p < 0.0001; 24.52 (8.86-67.90), p < 0.0001; AST: HR: 25.68 (7.49-88.07), p < 0.0001; 116.72 (31.58-431.46), p < 0.0001; TBIL: HR: 8.18 (4.03-16.61), p < 0.0001; 19.56 (5.21-73.44), p < 0.0001

Limitations

The present systematic review has certain limitations worth noting. First, the observational character of all included studies may impose a danger of bias in data collection. Second, few studies did not exclude patients with pre-existing liver conditions, making them vulnerable to more profound alterations in LFTs and a higher risk of detrimental outcomes. Lastly, papers that were not published in the English language were excluded even though they might have been relevant to this systematic review.

## Conclusions

This systematic review investigated the impact of SARS-CoV-2-related hepatic dysfunction on the clinical outcome of COVID-19. We concluded that multiple organ systems could be negatively affected by the SARS-CoV-2 virus, through its interaction with widely expressed ACE2R throughout the human body. The etiology of hepatic dysfunction in COVID-19 is multifactorial. Abnormal liver function tests are common in patients infected with the SARS-CoV-2 virus. They are significantly associated with mortality, higher frequency of ICU admission, and the need for mechanical ventilation. Therefore, it is important to closely follow up on hepatic function in COVID-19 patients.
